# The Role of Cancer Organoids in Ferroptosis, Pyroptosis, and Necroptosis: Functions and Clinical Implications

**DOI:** 10.3390/biom15050659

**Published:** 2025-05-02

**Authors:** Dingci Lu, Bingqian Xia, Tianquan Feng, Gui Qi, Zhaowu Ma

**Affiliations:** 1 The First Affiliated Hospital of Yangtze University, Yangtze University, Nanhuan Road 1, Jingzhou 434023, China; 2022710975@yangtzeu.edu.cn (D.L.); 2024711029@yangtzeu.edu.cn (B.X.); tqfeng2@126.com (T.F.); 2 School of Basic Medicine, Health Science Center, Yangtze University, Nanhuan Road 1, Jingzhou 434023, China; 3 Department of Clinical Laboratory, The Second Hospital of Jingzhou, Jingzhou 434000, China

**Keywords:** organoid models, ferroptosis, pyroptosis, necroptosis, cancer research, personalized medicine

## Abstract

The enduring prevalence of cancer worldwide constitutes a significant public health challenge, thereby emphasizing the imperative for the development of therapeutic models capable of accounting for the heterogeneity inherent in tumors. In this context, cancer organoids have emerged as powerful tools for studying tumor biology, providing valuable insights into the complex interactions within the tumor microenvironment. Concurrently, research is increasingly focused on non-apoptotic forms of regulated cell death (RCD)—including ferroptosis, pyroptosis, and necroptosis—which exert pivotal influences on cancer development and progression. Cancer organoids not only recapitulate the genetic and phenotypic heterogeneity of the original tumors but also enable more precise investigations into the roles of non-apoptotic RCDs within oncology. This review explores the utility of cancer organoids in delineating the molecular mechanisms underlying RCDs and their implications for cancer biology and treatment responses. By synthesizing recent research findings, it highlights the essential role of organoid models in uncovering the intricate details of non-apoptotic RCDs. Furthermore, it emphasizes promising directions for future research that aim to deepen our understanding of these pathways and their therapeutic potential. The integration of organoid models into investigations of ferroptosis, pyroptosis, and necroptosis provides novel insights into oncogenic mechanisms and facilitates the development of targeted therapeutic strategies. By bridging cancer organoids with human pathophysiology, this approach not only provides a transformative framework for dissecting oncogenic pathways but also enables the design of precision therapeutics that selectively target the molecular machinery underlying non-apoptotic RCDs.

## 1. Introduction

The global cancer burden causes nearly 10 million deaths annually and is rising, with effective treatment still a major unmet need due to tumor heterogeneity complicating therapy predictions, highlighting the urgent need for reliable models of the tumor microenvironment. In this context, organoid models have become a breakthrough in biomedical research, offering a significant advancement over traditional two-dimensional (2D) cell cultures [1]. These three-dimensional (3D) structures, derived from stem cells, self-organize into complex, organ-like architectures that closely mimic both the physiological and pathological conditions of human organs [2]. The advent of organoid technology has opened new avenues for studying various biological processes, including cancer biology, drug screening, and personalized medicine [3]. This advancement is crucial for tackling tumor heterogeneity and improving our understanding of the tumor microenvironment, aiding in more effective therapies. Given their ability to replicate complex tumor interactions, organoid models serve as an ideal platform to explore how dysregulated cell death pathways contribute to cancer progression.

Cell death has been associated with a variety of conditions arising from the dysregulation or malfunction of cell death signaling pathways [4]. This phenomenon can be classified into two primary categories: accidental cell death, which occurs as a result of uncontrolled biological processes triggered by unintentional injury stimuli, and regulated cell death (RCD), which is orchestrated by integrated signaling pathways and clearly defined mechanisms [5]. RCD can be further divided into apoptotic and non-apoptotic forms [6,7]. In the past decade, the most frequently investigated types of non-apoptotic RCD have encompassed ferroptosis, necroptosis, and pyroptosis [8,9]. The roles of non-apoptotic RCDs in cancer occurrence and development have gradually been revealed [10]. These RCDs play a crucial role not only in the growth and spread of cancer cells but also in influencing the effectiveness of cancer treatments [11]. Ferroptosis is a form of cell death induced by iron-mediated lipid peroxidation, which has gained widespread attention in cancer research in recent years. Its unique molecular mechanisms and roles in various cancer types make it a potential therapeutic target [12]. Pyroptosis is a form of programmed cell death mediated by inflammasomes, primarily executed through the cleavage of gasdermin family proteins [7]. The role of pyroptosis in cancer immunotherapy is also gradually being recognized. Necroptosis is a form of cell death mediated by receptor-interacting protein kinases (RIPK1 and RIPK3), and its role in cancer cannot be overlooked [13].

The integration of organoid models with the study of non-apoptotic RCD offers a promising approach to unraveling the complex interactions between these cell death pathways and cancer biology [14]. Organoids provide a more physiologically relevant platform to investigate how these forms of cell death influence tumor growth, metastasis, and response to therapies [15]. For instance, the use of colorectal cancer organoids has shed light on the role of ferroptosis in overcoming drug resistance [16]. Similarly, significant progress has been made in studying pyroptosis and necroptosis in pancreatic cancer and liver cancer [17]. The necessity and potential value of integrating organoid models with the exploration of these intricate cell death mechanisms reside in their remarkable capacity to unveil insights unattainable through traditional models [18].

Organoids can recapitulate biological heterogeneity associated with cancer patients, allowing for more accurate studies of how non-apoptotic RCD contributes to cancer biology and treatment outcomes [19]. They also facilitate the identification of novel therapeutic targets and the development of more effective cancer treatments [20]. This review provides a comprehensive analysis of the recent advancements in organoid technology, exploring the intricate biological mechanisms of cell death pathways and their relevance to oncological research. Moreover, organoids serve as a bridge between in vitro and in vivo models, offering a more physiologically relevant system for studying cancer progression and response to therapies. By mimicking the tumor microenvironment, they enable researchers to dissect the complex interactions between cancer cells and their surrounding stroma, which are crucial for understanding the role of non-apoptotic RCDs in cancer.

## 2. Cancer Organoids and Non-Apoptotic RCDs

### 2.1. Cancer Organoids

Organoid models have emerged as a revolutionary tool for studying complex biological processes, including the study of ferroptosis, pyroptosis, and necroptosis, which are forms of regulated cell death with significant implications for cancer research. The advent of cancer organoids as a model system heralds a remarkable evolution within the realm of oncology, presenting a refined alternative to conventional 2D cell cultures [21]. These small, self-organizing 3D constructs not only mirror the intricate cellular architecture of the tumor but also preserve the genetic and molecular signatures of the original cancerous tissue [22]. The technology harnesses the intrinsic plasticity of stem or progenitor cells extracted from tumor specimens, empowering them to differentiate into the diverse cell types that comprise the tumor mass and its surrounding microenvironment [3,23].

The cultivation of organoids typically necessitates a supportive 3D matrix, such as Matrigel or other extracellular matrix (ECM) components, which furnish the essential mechanical and biochemical signals for the cells to assemble into functional units [24]. This microenvironment is paramount, as it replicates the native tumor milieu, replete with the spatial organization and cell–cell interactions that are frequently compromised in 2D cultures [25].

A pivotal advantage of cancer organoids lies in their capacity to encapsulate the heterogeneity inherent within individual tumors. This includes both the inter- and intra-tumoral heterogeneity, which is reflective of the varied cellular constituents and microenvironments that are instrumental in shaping the phenotypic expression and behavioral characteristics of the tumor [15]. The preservation of this heterogeneity is vital for unraveling the complexities of cancer progression and for devising personalized treatment strategies tailored to the distinctive characteristics of a patient’s tumor [26].

Furthermore, cancer organoids facilitate the modeling of tumor–stroma interactions, which are indispensable for the growth, invasion, and metastasis of malignant cells [27]. The stroma, comprising fibroblasts, immune cells, and blood vessels, can be co-cultured with organoids to investigate the intricate crosstalk between the neoplastic cells and their surrounding microenvironment [28]. This interaction is crucial for the advancement of therapies aimed at disrupting the supportive role of the stroma in tumor maintenance and progression.

Another significant application of cancer organoids resides in the domain of pharmacology. They provide a platform for high-throughput drug screening and the evaluation of drug efficacy and toxicity within a preclinical context [29]. The patient-derived nature of organoids allows for the modeling of individual responses to treatment, thereby facilitating the identification of biomarkers that predict therapeutic outcomes and resistance [30]. Together, cancer organoids emerge as a formidable asset in the arsenal of cancer research, offering a more precise and physiologically relevant model for the exploration of cancer biology and for the development and testing of novel therapeutic strategies [31].

### 2.2. Non-Apoptotic RCDs

#### 2.2.1. Ferroptosis

Ferroptosis is an iron-dependent form of regulated cell death characterized by the accumulation of lipid peroxides to lethal levels [12]. The process is distinct from other forms of cell death, such as apoptosis and necrosis, and it involves key molecules and signaling pathways that regulate iron metabolism, lipid peroxidation, and antioxidant defenses [32]. One of the notable morphological features of ferroptosis is cell swelling accompanied by plasma membrane rupture. At its core, ferroptosis arises from the peroxidation of lipids associated with the cell membrane, which essentially means the excessive oxidative destruction of these lipids [33]. This destructive process heavily relies on several critical factors: reactive oxygen species (ROS), iron, and phospholipids that contain polyunsaturated fatty acids (PUFAs) [34]. Ferroptosis can be induced primarily through two distinct pathways—the transporter-dependent (extrinsic) pathway and the enzyme-regulated (intrinsic) pathway. In the transporter-dependent pathway, the inhibition of specific membrane transporters plays a key role. For example, the cystine–glutamate antiporter (system Xc−) [8], composed of family 7 member 11 (SLC7A11) and SLC3A2, is crucial for importing cystine into cells. When this system is inhibited, such as by erastin, cystine levels decrease, leading to reduced synthesis of glutathione (GSH). GSH is essential for the antioxidant defense system, and its depletion results in increased lipid peroxidation and ultimately ferroptosis [35]. In the enzyme-regulated pathway, specific enzymes within the cell play pivotal roles in initiating and propagating ferroptosis. One key enzyme is glutathione peroxidase 4 (GPX4), which directly reduces lipid hydroperoxides to non-toxic alcohol products, thereby preventing lipid peroxidation [36]. Inhibitors like RSL3 can directly inactivate GPX4 by reacting with its active site, selenocysteine, leading to the accumulation of lipid peroxides and ferroptotic cell death [16]. Another important enzyme is acyl-CoA synthetase long-chain family member 4 (ACSL4), which is involved in the synthesis of lipid substrates susceptible to peroxidation. The overexpression of ACSL4 increases the levels of PUFAs in cellular membranes, making cells more prone to ferroptosis [37]. Additionally, iron metabolism is tightly linked to ferroptosis, with iron overload promoting the Fenton reaction, which generates ROS that drive lipid peroxidation [33].

Ferroptosis is closely linked to cancer development and is implicated in cancer cell metabolic reprogramming. Tumor cells often exhibit altered iron metabolism, which can make them more susceptible to ferroptosis [38]. For example, research has underscored the promising therapeutic potential of ferroptosis in oncology by showing that a combination of luteolin and erastin can synergistically trigger ferroptosis in colon cancer cells. This approach adeptly circumvents erastin resistance through the targeted downregulation of the antioxidase GPX4 and the upregulation of the tumor suppressor hypermethylated in cancer 1, precisely exploiting the metabolic susceptibilities of these cancerous cells [39]. Similarly, pancreatic cancer cells with KRAS mutations exhibit increased vulnerability to ferroptosis due to elevated levels of ferroptosis suppressor protein 1 (FSP1), which protects against lipid peroxidation [40].

#### 2.2.2. Pyroptosis

Pyroptosis is a lytic programmed cell death mediated by gasdermin family proteins, characterized by inflammasome activation and pro-inflammatory cytokine release [7]. Pyroptosis occurs through canonical and non-canonical pathways. In the canonical pathway, pattern recognition receptors (PRRs) like NOD-like receptor thermal protein domain-associated protein 3 (NLRP3), AIM2, and NAIP/NLRC4 recognize pathogen-associated molecular patterns (PAMPs) or damage-associated molecular patterns (DAMPs), forming the inflammasome complex [41]. This complex activates caspase-1, which cleaves pro-IL-1β and pro-IL-18 into mature cytokines and processes gasdermin D (GSDMD) into GSDMD-N and GSDMD-C fragments. GSDMD-N inserts into the cell membrane, forming pores that disrupt membrane integrity, causing cell lysis and cytokine release [42]. In the non-canonical pathway, caspase-4/5 (in humans) or caspase-11 (in mice) directly recognize bacterial components, like LPS. Activated caspases cleave gasdermins (GSDMD, GSDME, or GSDMB), leading to pore formation, cell lysis, and the release of inflammatory contents [43].

Pyroptosis plays a dual role in cancer, acting as a tumor suppressor by eliminating malignant cells and as a promoter of tumor progression by creating a pro-inflammatory microenvironment that supports tumor growth and metastasis [44]. For instance, in colorectal cancer, the activation of pyroptosis has been shown to enhance anti-tumor immunity by releasing tumor antigens and pro-inflammatory cytokines that recruit immune cells to the tumor site [45]. Moreover, in breast cancer, the activation of pyroptosis leads to the release of high mobility group box 1 protein (HMGB1), which stimulates the TLR4 pathway in TAMs and facilitates the secretion of IL-1β, thereby supporting tumor progression and metastasis [46].

#### 2.2.3. Necroptosis

As a type of regulated necrosis distinct from apoptosis, necroptosis manifests unique cytological features including cytoplasmic vacuolization, disintegration of the plasma membrane, and extracellular release of cytoplasmic components [13]. Necroptosis is primarily regulated by RIPK1 and RIPK3 and the pseudokinase mixed lineage kinase domain-like protein (MLKL) [47]. Upon activation by death receptors, such as tumor necrosis factor receptor-1, RIPK1 interacts with RIPK3 to form the necrosome complex, which subsequently phosphorylates MLKL [48]. The phosphorylated MLKL undergoes conformational changes, exposing its N-terminal four-helix bundle domain and forming homo-oligomers. These oligomers translocate to the plasma membrane, where they bind to phosphatidylinositol phosphate species [10]. This binding leads to the disruption of membrane integrity, causing cell lysis and the release of damage-associated molecular patterns (DAMPs), which contribute to inflammation.

Necroptosis is strongly associated with cancer development and contributes to drug resistance in cancer. Tumor cells that evade apoptosis may become susceptible to necroptosis, providing an alternative pathway for cell death. For example, colorectal cancer cells resistant to apoptosis have been shown to upregulate RIPK1, making them more prone to necroptosis [49]. Similarly, targeting necroptosis pathways has been proposed as a strategy to overcome resistance to conventional therapies in various cancers [50].

Cancer organoid models enhance our understanding of tumor heterogeneity and non-apoptotic RCDs, serving as relevant platforms for oncology research and treatment response insights. Their role in elucidating cell death mechanisms, cancer biology, and treatment outcomes is increasingly recognized.

## 3. Various Cancer Organoid Models in Non-Apoptotic RCD

**Figure 1:**
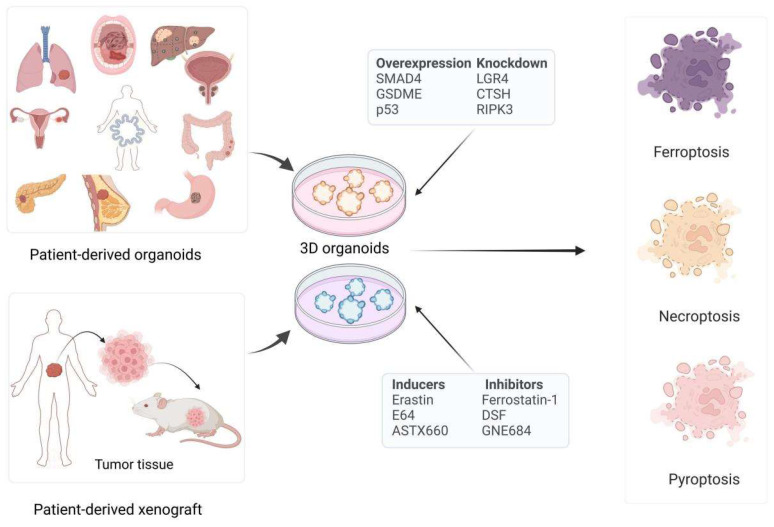
The emerging roles of cancer organoid models in non-apoptotic RCDs. The integration of cancer organoid models with non-apoptotic RCDs enhances applications in cancer therapy research. By deftly manipulating RCD-related genes within these organoids, one unveils intricate disease mechanisms and potential therapeutic targets. The application of RCD inhibitors and inducers within these organoid models delves into the pivotal role of cell death in both cancer progression and treatment efficacy. LGR4: G protein-coupled receptor 4; CTSH: cathepsin H; RIPK3 erastin: ferroptosis activator; E64: proteinase inhibitor E64; ASTX660: IAP antagonist tolinapant; ferrostatin-1: ferroptosis inhibitors; DSF: pyroptosis inhibitor; GNE684: RIP1 inhibitor. Created with BioRender.com.

Organoid models tailored to various cancer types have demonstrated immense potential in the study of non-apoptotic RCD pathways, such as ferroptosis, pyroptosis, and necroptosis. These models not only provide a more authentic simulation of the tumor microenvironment but also serve as a critical platform for understanding the mechanisms of cell death. Cancer cells often gain a survival advantage by evading these pathways [43], making a thorough investigation of the roles of ferroptosis, pyroptosis, and necroptosis in organoid models crucial for the development of novel cancer therapeutic strategies. In the following sections, we will summarize the applications of various cancer organoid models in the study of ferroptosis, pyroptosis, and necroptosis, as well as how they provide new insights and directions for anti-tumor treatment (Figure 1 and Table 1). Further analysis of the research outcomes derived from organoid models will be essential to fully elucidate their potential to advance cancer therapy strategies.

### 3.1. Ferroptosis in Cancer Organoid Models Research

#### 3.1.1. Ferroptosis in Breast Cancer Models Research

Emerging research on human breast cancer organoids has unveiled the potential of targeted therapies to induce ferroptosis, a novel approach to enhance tumor suppression and counteract drug resistance in breast cancer treatment. For example, studies utilizing human triple-negative breast cancer (TNBC) organoids have revealed that the concurrent inhibition of focal adhesion kinase (FAK) and ROS1 leads to a synergistic reduction in tumor proliferation, primarily through the upregulation of p53 signaling pathways and the induction of ferroptosis [51]. Furthermore, the combination of anti-fibroblast growth factor receptor 4 (FGFR4) and anti-HER2 therapies has been identified as a mechanism to trigger ferroptosis in HER2-positive breast cancer. Experimental evidence from patient-derived xenografts and organoid studies illustrates the synergistic benefits of this combination treatment, presenting a promising strategy to combat resistance in HER2-positive breast cancer cases [52]. Additionally, Tamoxifen, a widely recognized therapeutic agent for breast cancer, has been found to induce ferroptosis in MCF-7 breast cancer organoids. This finding highlights the potential of ferroptosis induction to enhance the effectiveness of current breast cancer treatments and to mitigate drug resistance [53].

#### 3.1.2. Ferroptosis in Pancreatic Cancer Models Research

Pancreatic cancer, particularly pancreatic ductal adenocarcinoma (PDAC), presents significant challenges in treatment due to its aggressive characteristics and inherent resistance to standard therapeutic approaches. For instance, an investigation has identified the potential of small molecule chimeras, including derivatives of salinomycin and dihydroartemisinin, in triggering ferroptosis within drug-tolerant PDAC cells and organoids [20]. Moreover, the mitochondrial calcium uniporter (MCU) has emerged as a significant contributor to metastasis and represents a targetable weakness for inducing ferroptosis in pancreatic cancer. Pharmacological blockade of the cystine transporter solute carrier SLC7A11/xCT has demonstrated effectiveness in promoting tumor regression and inhibiting MCU-mediated metastasis in organoid models derived from patients [54]. Consequently, organoid models serve as a robust platform for elucidating these mechanisms and pinpointing prospective therapeutic targets. Focusing on critical regulators, such as FSP1, MCU, and SMAD4, along with leveraging the vulnerabilities linked to ferroptosis, presents a promising avenue for enhancing treatment outcomes in patients with pancreatic cancer.

Organoid models originating from pancreatic cancer have yielded significant insights into the implications of ferroptosis in cancer treatment. Notably, increased levels of FSP1 have been identified as a protective mechanism for KRAS-mutated cells against ferroptosis during the early stages of tumor development, indicating a promising target for therapeutic strategies [40]. Furthermore, organoids derived from patients have illustrated that the modulation of the macrophage-capping protein (MCP)-GPX4/HMGB1 pathway can instigate immunogenic ferroptosis, thereby presenting a novel strategy to bolster anti-tumor immune responses [55]. Moreover, the combination of gemcitabine with ferroptosis inducers has demonstrated an enhancement of cytotoxic effects in SMAD4-positive organoids, proposing a viable combination therapy for pancreatic cancer [56].

#### 3.1.3. Ferroptosis in Liver Cancer Models Research

Recent advancements in hepatocellular carcinoma (HCC) research utilizing patient-derived organoids have shed light on the potential of targeted therapeutic combinations to induce ferroptosis, a promising strategy for enhancing treatment outcomes and overcoming drug resistance in liver cancer. Research involving patient-derived organoids has revealed that the combination of donafenib and GSK-J4 effectively promotes ferroptosis in liver cancer through the upregulation of heme oxygenase (HMOX1) expression, indicating a promising avenue for combination therapy [57]. Moreover, organoid models sourced from HCC patients have illustrated that metformin can reactivate peroxisome proliferator-activated receptor-gamma coactivator-1α (PPARGC1A) expression and augment ferroptosis, underscoring its relevance in the pathogenesis of HCC and potential therapeutic strategies [58]. Additionally, studies employing HCC organoids have identified that the unconventional prefoldin RPB5 interactor (URI) plays a role in mediating resistance to ferroptosis induced by tyrosine kinase inhibitors (TKIs), thereby presenting a potential target for addressing drug resistance. The synergistic application of the stearoyl-CoA desaturase 1 (SCD1) inhibitor aramchol alongside the deuterated sorafenib derivative donafenib has shown significant anti-tumor efficacy in p53-wild type HCC organoids [59].

Furthermore, the integration of ferroptosis induction with alternative therapeutic modalities, such as the inhibition of myeloid-derived suppressor cells (MDSCs), has increased the susceptibility of both primary tumors and liver metastases to immune checkpoint inhibitors, thereby presenting a novel paradigm in cancer immunotherapy. This investigation underscores the potential of immune responses elicited by ferroptosis in the management of primary liver tumors and metastatic liver lesions, although it does not exert the same influence on colorectal cancer (CRC) organoids in subcutaneous settings, while it does diminish their metastatic proliferation in the liver [60]. The merging of ferroptosis research and organoid models presents a promising opportunity in liver cancer research.

#### 3.1.4. Ferroptosis in Gastric Cancer Models Research

Organoid models have been employed to investigate the therapeutic potential of ferroptosis in the context of gastric cancer. Research has demonstrated that cancer-associated fibroblasts (CAFs) hinder the cytotoxic capabilities of natural killer (NK) cells in gastric cancer by promoting ferroptosis through iron modulation. This mechanism was clarified using a human patient-derived organoid model, wherein the simultaneous targeting of CAFs with deferoxamine and a follistatin–like protein 1 neutralizing antibody significantly mitigated the CAF-induced ferroptosis in NK cells and enhanced their cytotoxic activity against gastric cancer cells [61]. Furthermore, the inhibition of the activator of the transcription 3 (STAT3)–ferroptosis regulatory pathway presents a promising therapeutic avenue for addressing chemotherapy resistance and improving treatment outcomes in gastric cancer. Specifically, the targeting of STAT3 using W1131 has been shown to induce ferroptosis, exhibiting notable anti-tumor effects across various experimental models, including organoids and patient-derived xenografts [62].

#### 3.1.5. Ferroptosis in Colorectal Cancer Models Research

CRC has been the subject of extensive research utilizing organoid models to elucidate the mechanisms underlying ferroptosis and its therapeutic implications. Organoids derived from CRC have exhibited potential in mitigating acquired drug resistance through the induction of ferroptosis. For example, research indicates that the absence of Hmox1 in colonic epithelial cells facilitates ferroptosis, thereby presenting a viable target for therapeutic strategies [63]. Furthermore, organoids derived from intestinal stem cells have underscored the significance of IFNγ as a pivotal cytokine that can halt cancer stemness and initiate GPX4-dependent ferroptosis [64]. Additionally, patient-derived organoids have been employed to demonstrate that compounds, such as curcumin and andrographis, possess anti-tumor properties by activating ferroptosis [65]. In addition, CRC organoids have illustrated that exosomes derived from adipocytes, which contain microsomal triglyceride transfer protein (MTTP), diminish susceptibility to ferroptosis in CRC, thereby enhancing chemoresistance to oxaliplatin [66]. Collectively, these findings highlight the potential of targeting ferroptosis as a therapeutic avenue in the treatment of CRC.

Organoid models have illuminated the intricacies of ferroptosis in CRC, positioning it as a promising therapeutic avenue. Investigations have revealed that interventions targeting key signaling cascades, including G protein-coupled receptor 4 (LGR4) and the β-catenin/Wnt pathway, can re-sensitize CRC to chemotherapeutic agents by triggering ferroptosis, thereby countering drug resistance [16]. A study shows that andrographis, a plant-based compound, improves the effectiveness of chemotherapy in 5FU-resistant CRC by inducing ferroptosis and altering β-catenin/Wnt signaling. This is supported by its combined effects observed in both cell lines and patient-derived organoids [67]. Moreover, the combined use of Vitamin C and cetuximab has shown promising results in CRC organoid models by activating ferroptosis, a synthetic lethal metabolic cell death pathway, to prevent the development of drug resistance and delay the emergence of persister cells, potentially offering a clinical strategy for enhancing the efficacy of anti-EGFR therapies [68].

#### 3.1.6. Ferroptosis in Ovarian Cancer Models Research

Organoid models have significantly advanced our understanding of the implications of ferroptosis in ovarian cancer (OC). In this context, the metabolic processes involving lipids and the redox-dependent mechanism of ferroptosis are modulated by fatty acid desaturases, specifically SCD1 and acyl-CoA 6-desaturase (FADS2). These enzymes play a crucial role in regulating lipid metabolism and ferroptosis, thereby affecting the survival and proliferation of cancer cells. For example, organoids derived from high-grade serous ovarian cancer (HGSOC) have demonstrated that the application of iron nitroprusside (FeNP) inhibits GPX4 activity, which subsequently triggers ferroptosis, indicating a potential avenue for therapeutic intervention [69]. Furthermore, the fatty acid desaturases SCD1 and FADS2 have been implicated in maintaining a balance between lipid metabolic activity and redox-driven ferroptosis in ovarian cancer cells sourced from ascites. The strategic targeting of lipid metabolic pathways to promote ferroptosis appears to hold promise in addressing resistance to standard therapeutic regimens [70].

#### 3.1.7. Ferroptosis in Bladder Cancer Models Research

Organoid models have also been utilized to explore the role of ferroptosis in bladder cancer (BCa). A recent investigation revealed that phosphoglycerate dehydrogenase (PHGDH) enhances the expression of SLC7A11, a vital component of the System Xc-, which is essential for sustaining GSH levels and inhibiting ferroptosis. Subsequent functional assays demonstrated that the PHGDH inhibitor NCT-502 prompted ferroptosis in BCa cell organoid models, resulting in diminished tumor growth [66]. Additionally, N6-methyladenosine (m6A) modifications play a role in chemoresistance by modulating the RNA stability and protein expression of SLC7A11, thereby affecting the sensitivity of bladder cancer cells to ferroptosis. This mechanism is rapidly activated in both cisplatin-sensitive cell lines and patient-derived organoids following brief exposure to cisplatin, indicating a common pathway involving SLC7A11 upregulation and chemoresistance [71].

#### 3.1.8. Ferroptosis in Other Cancer Models Research

Recent findings derived from patient-derived organoid models underscore the promising therapeutic implications of targeting ferroptosis across various malignancies. Specifically, organoids obtained from cholangiocarcinoma patients have revealed that the combination of surufatinib (SUR) and photodynamic therapy (PDT) not only triggers ferroptosis but also suppresses tumor proliferation [72]. In the context of glioblastoma, patient-derived organoid models have illustrated that a combinatorial treatment approach can potentiate ferroptosis by modulating the expression of HOXM1 and GPX4 [73]. Likewise, organoids from oral squamous cell carcinoma have demonstrated that the inhibition of dynamin-related protein 1 (DRP1), which leads to mitochondrial elongation, facilitates ferroptosis and diminishes cancer stemness characteristics [42]. Additionally, in lung organoid models, mannose glycosides have been shown to enhance lung cancer cell sensitivity to EGFR-TKI therapy via the induction of ferroptosis [74]. Similarly, in organoid models of prostate cancer and laryngeal squamous cell carcinoma, activating the androgen receptor (AR)/GPX4 axis and inhibiting endoplasmic reticulum oxidoreductase 1 alpha (ERO1α) have both led to improved therapeutic outcomes [75,76]. In the realm of head and neck cancer, organoid models have played a crucial role in revealing the synergistic effects of targeting thioredoxin reductase 1 (TrxR1) inhibition in conjunction with anti-PD-1 therapy, thereby emphasizing the potential of ferroptosis induction to enhance therapeutic outcomes in cancer treatment [77]. Overall, the integration of organoid models with ferroptosis research not only clarifies the significance of ferroptosis in drug-tolerant persister cells but also opens new pathways for innovative cancer therapies.

### 3.2. Pyroptosis in Cancer Organoid Models Research

Cancer organoid models have emerged as invaluable and revolutionary tools for studying the intricate and complex mechanisms of pyroptosis, a form of programmed cell death characterized by inflammatory responses, in various cancer types, providing researchers with a more accurate representation of tumor biology and enabling them to explore the nuances of cellular interactions and therapeutic responses in a controlled environment.

In PDAC, organoid models have been instrumental in elucidating the role of pyroptosis. Pyroptosis-based therapies show promise in tackling chemoresistance in pancreatic and lung cancers. The suppression of chemotherapy-induced pyroptosis by β5-integrin, facilitated by sphingolipid metabolic enzyme ceramidase-driven sphingolipid metabolism, emerges as a critical mechanism. This suppression can be overcome with Src or ceramidase inhibitors, restoring pyroptosis and enhancing chemotherapy response in vitro and in vivo, demonstrated through experiments with cancer cell lines, patient-derived tumor organoids, and orthotopic animal models [44].

Beyond PDAC, organoid models have illuminated the intricacies of pyroptosis in diverse cancer types. In colorectal cancer, organoid-based drug screening has identified small molecule inhibitors that induce concurrent apoptosis and gasdermin E-dependent pyroptosis, highlighting the potential of these models in discovering novel therapeutic targets [78]. Studies utilizing colonic epithelial organoids have further revealed EGFR inhibition-induced chromosomal instability and altered pyroptosis genes, linking pyroptosis to early cancer development [45].

Bladder cancer organoids have also provided insights into the mechanisms of pyroptosis. A study revealed that chemotherapy-induced differentiation in bladder cancer organoids could trigger pyroptosis, suggesting a potential therapeutic strategy for enhancing treatment efficacy [79]. In endometrial cancer, organoid models have been used to explore the role of estrogen-related receptor alpha (ERRα) in promoting glycolytic metabolism and targeting the NLRP3/caspase-1/GSDMD pathway to regulate pyroptosis, providing a deeper understanding of the metabolic and inflammatory pathways involved in cancer [80].

Furthermore, organoid models of esophageal adenocarcinoma have been utilized to characterize the involvement of caspase-1 in disease progression, shedding light on the molecular mechanisms underlying pyroptosis in this cancer type [81]. In Her2-positive gastric cancer, the use of anti-PD-1/Her2 bispecific antibodies in organoid models has demonstrated the induction of gasdermin B-cleavage and pyroptosis, offering promising therapeutic avenues [82]. Ovarian cancer organoids have also been employed to study the immunoreactive microenvironment and the role of guanylate-binding protein 5 (GBP5) in suppressing cancer progression through canonical pyroptosis [83]. Omental adipocyte pyroptosis, mediated by IL-6 and IL-8 from OC cells, triggers free fatty acids and ATP release, enhancing chemoresistance and macrophage infiltration in advanced OC, which is modeled in vitro using organoids and validated in vivo, suggesting pyroptosis inhibition as a therapeutic approach [84].

In conclusion, cancer organoid models have significantly advanced our understanding of pyroptosis in various cancer types. These models establish reproducible platforms for investigating the molecular mechanisms of pyroptosis, identifying potential therapeutic targets, and developing novel treatment strategies.

### 3.3. Necroptosis in Cancer Organoid Models Research

By utilizing organoids, researchers can better mimic the tumor microenvironment, allowing for a more accurate study of necroptosis and its effects on cancer progression and treatment responses. This innovative approach not only enhances our comprehension of the mechanisms underlying necroptosis but also opens new avenues for developing targeted therapies that could improve patient outcomes.

In PDAC, organoid models have been used to study the involvement of necroptosis in cancer progression and treatment resistance. A study demonstrated that PDAC organoids could recapitulate the tumor microenvironment, allowing for the investigation of necroptosis pathways and their therapeutic implications [85].

Colorectal cancer organoids have also been instrumental in studying necroptosis. A study highlighted the clinical positioning of the inhibitors of apoptosis protein antagonist tolinapant (ASTX660) in colorectal cancer, demonstrating its ability to induce necroptosis and overcome treatment resistance [86]. Additionally, organoid models have been used to explore the regulation of RIPK1 by the fragile X mental retardation protein (FMRP) and its role in colorectal cancer resistance to necroptosis [49].

In the context of graft-versus-leukemia (GVL), organoid models have provided insights into the role of RIP1 inhibition in improving immune reconstitution and reducing graft-versus-host disease (GVHD) mortality while preserving GVL effects [50]. This highlights the potential of targeting necroptosis pathways to enhance therapeutic outcomes in hematological malignancies.

Furthermore, studies on mutant p53-expressing cells have utilized organoid models to investigate the role of necroptosis in cell competition and tumor suppression. It was found that mutant p53-expressing cells undergo necroptosis via cell competition with neighboring normal epithelial cells, providing a novel perspective on tumor suppression mechanisms [87]. In gastric cancer, organoid models have been used to study the role of interleukin-17A in promoting parietal cell atrophy through apoptosis and necroptosis, offering insights into the inflammatory pathways involved in cancer progression [88].

Overall, cancer organoid models have proven to be invaluable tools for studying necroptosis in various cancer types. These models enable the investigation of the molecular mechanisms underlying necroptosis, the identification of potential therapeutic targets, and the development of novel treatment strategies. The integration of organoid models in necroptosis research holds great promise for advancing our understanding of cancer biology and improving therapeutic outcomes.

## 4. Therapeutic Implications of Non-Apoptotic RCD in Cancer Organoids

**Figure 2:**
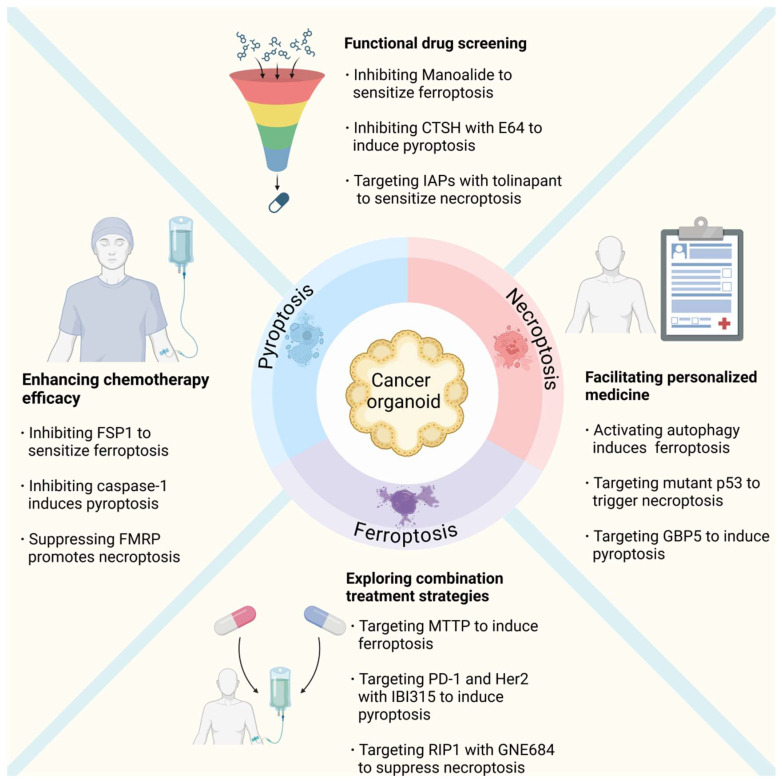
Combining cancer organoids and non-apoptotic RCDs in therapeutic strategies. Organoid models in cancer research highlight the role of functional drug screening, enhancing chemotherapy efficacy by targeting specific cell death pathways, exploring combination treatment strategies, and facilitating personalized medicine through tailored treatment strategies. These advanced applications are reshaping the landscape of cancer therapy, offering more precise and effective approaches. E64: proteinase inhibitor E64; IAPs: Inhibitors of apoptosis proteins; FSP1: ferroptosis suppressor protein 1; FMRP: fragile X mental retardation protein; GBP5: guanylate-binding protein 5; MTTP: microsomal triglyceride transfer protein; IBI315: anti-PD-1/Her2 bispecific antibody; GNE684:RIP1 inhibitor. Created with BioRender.com.

Organoid models offer a unique platform for studying the therapeutic implications of ferroptosis, pyroptosis, and necroptosis in cancer research. Their ability to closely mimic the architecture and microenvironment of human tissues enables functional drug screening, enhances chemotherapy efficacy, explores combination treatment strategies, and facilitates personalized medicine (Figure 2).

### 4.1. Functional Drug Screening

In drug screening, organoid models enable the high-throughput identification of compounds that can modulate ferroptosis, pyroptosis, and necroptosis, paving the way for the development of targeted therapies tailored to individual tumors. The organoid model has emerged as a pivotal tool in the discovery and testing of new drugs that target specific cell death pathways, providing a more personalized and effective approach to cancer treatment. For instance, in lung cancer research, organoids have been instrumental in drug screening, leading to the discovery of Manoalide (MA) as an agent that sensitizes KRAS-mutated and osimertinib-resistant lung cancer to EGFR-TKIs. By triggering ER stress and ferroptosis through an ROS-dependent mechanism, MA enhances the susceptibility of these cancer cells to osimertinib [74]. In a comparable manner, with respect to bladder cancer [79], organoids were used to screen treatments for inducing semi-squamatization, revealing that inhibiting cathepsin H with proteinase inhibitor E64 can trigger pyroptosis in chemoresistant muscle-invasive bladder cancer (MIBC) cells, suggesting a strategy to combat resistance. Additionally, the application of organoids in the context of CRC has exemplified targeted drug screening for necroptosis, demonstrating that the ASTX660 induces caspase-8-dependent apoptosis and, when combined with fluorouracil plus leucovorin and oxaliplatin (FOLFOX) chemotherapy, enhances therapeutic impact through the downregulation of class I histone deacetylases (HDACs) [86]. These studies underscore the utility of organoid models in drug screening to develop personalized cancer therapies that exploit specific cell death pathways.

### 4.2. Enhancing Chemotherapy Efficacy

Improving the effectiveness of chemotherapy is another significant application of organoid models in the context of these cell death mechanisms. Recent studies have underscored the potential of organoids to improve the effectiveness of chemotherapy by leveraging specific modes of cell death in cancer treatment [89]. In a phase III trial involving patients with locally advanced rectal cancer, tumor tissues from these patients were used to create organoids, which were found to closely mirror the patients’ own responses to chemoradiation treatment [90]. Organoids in pancreatic cancer research reveal drug resistance mechanisms, with FSP1 overexpression linked to ferroptosis resistance. Combining chemotherapy and FSP1 inhibitors increases KRAS-mutated cells’ susceptibility to ferroptosis, enhancing treatment efficacy [40]. In esophageal adenocarcinoma research, inhibiting the enzyme caspase-1, which is crucial for pyroptosis, in organoid models significantly reduces the secretion of cytokines that drive disease progression. This suggests a novel method to enhance chemotherapy by inducing pyroptosis [81]. Furthermore, in colorectal cancer organoid models, the suppression of FMRP, which regulates necroptosis through its interaction with RIPK1 mRNA, led to spontaneous cell death, suggesting that modulating FMRP could promote necroptosis and improve chemotherapy outcomes in chemoresistant cells [49].

### 4.3. Exploring Combination Treatment Strategies

Organoid models are instrumental in enhancing therapeutic synergy within combination cancer treatments, effectively targeting cell death pathways such as ferroptosis, pyroptosis, and necroptosis. To illustrate, in the context of CRC [66], researchers used organoids to pinpoint a combination therapy targeting MTTP to sensitize cells to ferroptosis, thereby enhancing the efficacy of CRC treatments. Similarly, in Her2-positive gastric cancer [82], organoids revealed that the anti-PD-1/Her2 bispecific antibody IBI315 could induce pyroptosis and activate T cells, creating a positive feedback loop for tumor cell killing. Additionally, in GVL, the inhibition of RIP1 mitigates the severity of graft-versus-host disease by reducing inflammation and enhancing immune reconstitution while preserving the protective effect against leukemia. This strategy enhances therapeutic synergy, offering a potential non-immunosuppressive treatment option for GVHD patients [50].

### 4.4. Facilitating Personalized Medicine

Organoid models play a pivotal role in facilitating personalized medicine by providing a means to evaluate the individual susceptibility of tumors to RCD pathways such as ferroptosis, pyroptosis, and necroptosis. In the context of HCC, research has demonstrated that the activation of autophagy can modulate m6A modification, thereby promoting ferroptosis. The findings indicate that the inhibition of either Wilms’ tumor 1-associating protein or YTH domain containing 2 can effectively halt ferroptosis and suppress the progression of HCC. This suggests that targeting specific epigenetic modifications to induce ferroptosis could provide a personalized therapeutic approach for HCC patients [91]. New research indicates that cells with mutant p53 can trigger a specialized cell death process, necroptosis, when in contact with normal cells, aiding in the early removal of potentially oncogenic cells and advancing personalized medicine. Understanding the sequence of p53 mutations is crucial as it can influence cancer progression and dictate treatment responses, thereby enhancing personalized therapeutic strategies. By integrating these findings with organoid model research, we can refine treatments, particularly for cancers like HCC, where p53 mutations are common and may lead to resistance against traditional therapies [87]. This approach ensures that therapeutic interventions are tailored to the specific molecular and cellular features of an individual’s cancer, potentially enhancing treatment efficacy. Similarly, in ovarian cancer, organoid models have been crucial in identifying GBP5 as a key factor in pyroptosis, a form of RCD that can inhibit tumor progression [83]. GBP5 was found to induce pyroptosis through the JAK2/STAT1 pathway in patient-derived organoids, offering a new avenue for targeted therapies. Therefore, by harnessing patient-derived organoids, researchers are poised to unveil biomarkers indicative of sensitivity to regulated cell death, thus facilitating the formulation of bespoke treatment regimens. This paradigm of precision medicine aspires to enhance patient outcomes while mitigating adverse effects, heralding a new era in personalized cancer therapeutics.

In conclusion, organoid models emerge as a formidable platform for exploring the therapeutic implications of ferroptosis, pyroptosis, and necroptosis in cancer research, offering groundbreaking applications in functional drug screening, enhancing chemotherapy efficacy, exploring combination treatment strategies, and facilitating personalized medicine. These models not only provide new insights into the intricate mechanisms of cell death but also enable the discovery of effective cell death inducers, thereby revolutionizing cancer treatment strategies. With the ongoing advancements in organoid technology, its potential applications in cancer research and therapy are anticipated to broaden and deepen, ushering in a new era of precision medicine.

## 5. Discussion

Organoid models have emerged as revolutionary tools in cancer research, providing a physiologically relevant platform for studying the complex mechanisms of non-apoptotic RCDs. This review highlights the significant advantages of these models in investigating ferroptosis, pyroptosis, and necroptosis, serving as powerful instruments for advancing our understanding of cancer biology and developing therapeutic strategies. By precisely replicating the tumor microenvironment and intricate cell–cell interactions in vitro, organoid models closely mimic the heterogeneity and pathophysiological processes of tumors. They offer a robust platform for drug screening and molecular mechanism research and facilitate the development of novel therapeutic approaches targeting specific cell death pathways. Furthermore, patient-derived organoids enable personalized medicine, allowing for the assessment of tumor sensitivity to treatment and the customization of more effective therapeutic regimens for individual patients. The advantages of cancer organoid models not only enhance the clinical relevance of research but also offer new possibilities for overcoming drug resistance and improving treatment outcomes, paving new avenues and hope for future cancer therapies.

Cancer organoids present a remarkable platform for the exploration of non-apoptotic RCDs, adeptly mirroring the biological heterogeneity intrinsic to primary tumors, thereby serving both basic research and translational medicine. However, there are a series of key questions that remain to be answered. (i) Are the expansion efficiency and genetic stability of organoids sufficient to support their application in non-apoptotic RCD research? Currently, the expansion efficiency and genetic stability of organoids are not yet fully sufficient to robustly support their application in non-apoptotic RCD research, necessitating further studies to optimize culture conditions and select more appropriate cancer subtypes. Nonetheless, organoids have demonstrated relatively good genetic stability in certain scenarios [92], offering promise for their use in non-apoptotic RCD research. (ii) Can organoid models in non-apoptotic RCD research adequately simulate the 3D culture environment and the complete tumor microenvironment to support their application in this field? Organoid models in non-apoptotic RCD research are limited by the ECM and cellular diversity, which may affect the simulation of cellular homeostasis and non-apoptotic RCD processes. To enhance research accuracy, future studies should integrate immune and stromal cells into organoid culture and optimize the ECM to better mimic the tumor microenvironment [21]. (iii) Standardized operations and reproducibility in cancer organoid culture could accelerate the advancement of research on non-apoptotic RCD. Changes due to long-term culture and the lack of assessment tools may compromise the stability and reliability of non-apoptotic RCD research. Consistent organoid culture conditions are necessary for researchers to accurately compare cell death patterns under different conditions. Establishing a standardized protocol for organoid culture enhances experimental reproducibility and provides researchers with a more precise and reliable research platform [93]. (iv) How can we overcome the limitations of culture efficiency and cost in non-apoptotic RCD research using organoids? The development of cost-effective media formulations and culture conditions is urgently needed to improve cancer organoid efficiency and reduce costs in non-apoptotic RCD research. Therefore, optimizing culture conditions and minimizing dependence on costly components can further reduce costs and enhance culture efficiency [15].

Looking ahead, the integration of cancer organoid technology with cutting-edge techniques offers a novel perspective for studying non-apoptotic RCD, heralding a new chapter in cancer research and treatment. The application of CRISPR-Cas9 technology to organoids [79] allows researchers to precisely edit genes, model diseases, and investigate the functions of genes in non-apoptotic RCD and cancer progression. By combining with omics technologies, such as single-cell RNA sequencing, a deeper understanding of the molecular mechanisms of cell death can be achieved [16]. The convergence of organoid technology with emerging techniques such as ECM scaffolds, microfluidic methods, and bioprinted 3D model systems holds immense potential to more comprehensively simulate the tumor microenvironment and could profoundly influence our understanding of cell death mechanisms [27,94,95]. In addition, the integration of cancer organoid technology with microbiota research and nanoparticle-based therapies provides a comprehensive approach to understanding the complex interactions between tumor cells, microbiota, and the host immune system and helps develop personalized treatment strategies leveraging non-apoptotic RCD [96,97,98].

## 6. Conclusions

Conclusively, cancer organoids have significantly enhanced the capability to investigate RCDs in oncology. However, the majority of studies have been conducted in preclinical models. Nevertheless, the prospects are encouraging, and there are several clinical trials registered on ClinicalTrials.gov involving organoids for RCD (NCT06435689, NCT00280202, NCT04549727). This development underscores the growing interest in translating preclinical findings into clinical applications. Therefore, further exploration of RCD mechanisms within cancer organoids may enhance the safety and selectivity of treatments. This could contribute to the development of more effective and targeted cancer therapeutic strategies.

## Figures and Tables

**Table 1:** Cancer organoid models in non-apoptotic RCDs.

Cancer Types	Organoid Models	Functions	Reference
**Ferroptosis**			
Breast cancer	Human TNBC organoid models	Simultaneous inhibition of FAK and ROS1 synergistically repress tumor growth by upregulating p53 signaling and inducing ferroptosis	[51]
Breast cancer	Breast cancer organoids	Combining anti-FGFR4 and anti-HER2 therapies induces ferroptosis in HER2-positive breast cancer	[52]
Breast cancer	MCF-7 organoid models	Tamoxifen can induce ferroptosis in MCF-7 organoid models	[53]
Pancreatic cancer	PDAC organoids	Iron-sensitive prodrugs that trigger active ferroptosis in drug-tolerant pancreatic cancer cells	[20]
Pancreatic cancer	Patient-derived organoids	Imidazole ketone erastin induces tumor regression and abrogates MCU-driven metastasis	[54]
Pancreatic cancer	Pancreatic organoids derived from a mouse model	Elevated FSP1 protects KRAS-mutated cells from ferroptosis during tumor initiation	[40]
Pancreatic cancer	Patient-derived organoids	Triggers immunogenic ferroptosis by targeting the MCP-GPX4/HMGB1 axis	[55]
Pancreatic cancer	SMAD family member 4 (SMAD4)-positive organoids	Enhances cytotoxic effects by combining gemcitabine with ferroptosis inducers	[56]
Liver cancer	Patient-derived organoids	Donafenib and GSK-J4 synergistically induced ferroptosis in liver cancer by upregulating HMOX1 expression	[57]
Liver cancer	Organoid models derived from HCC patients	Metformin restores PPARGC1A expression and enhances ferroptosis	[58]
Liver cancer	HCC organoids	URI mediates resistance to TKI-induced ferroptosis	[59]
Liver cancer	CRC organoids	Combining ferroptosis induction with MDSC blockade rendered primary tumors and metastases	[60]
Gastric cancer	Patient-derived organoid model	CAFs impair the cytotoxic function of NK cells in gastric cancer by inducing ferroptosis via iron regulation	[61]
Gastric cancer	Patient-derived organoids	Inhibition of the STAT3–ferroptosis negative regulatory axis suppresses tumor growth and alleviates chemoresistance	[62]
Colorectal cancer	Colonic epithelial organoids	Role in colitis-associated cancer, modulation of Hmox1 and ferroptosis	[63]
Colorectal cancer	Intestinal stem cell organoids	IFNγ synergies with cold atmospheric plasma to trigger ferroptosis	[64]
Colorectal cancer	Patient-derived organoids	Curcumin and andrographis exhibit anti-tumor effects via the activation of ferroptosis	[65]
Colorectal cancer	CRC organoids	Adipocyte-derived exosomal MTTP suppresses ferroptosis and promotes chemoresistance	[66]
Colorectal cancer	CRC-derived organoid	Overcome drug resistance through LGR4 targeting, enhance sensitivity to drug-induced ferroptosis	[16]
Colorectal cancer	Patient-derived organoids	Andrographis-mediated chemosensitization in CRC via the activation of ferroptosis	[67]
Colorectal cancer	CRC organoids	VitC disrupted iron homeostasis and increased ROS levels, ultimately leading to ferroptosis	[68]
Ovarian cancer	Ovarian cancer organoids derived from HGSOC	FeNP inhibits GPX4 activity, leading to the induction of ferroptosis	[69]
Ovarian cancer	Ovarian cancer organoids	SCD1/FADS2 fatty acid desaturases equipoise lipid metabolic activity and redox-driven ferroptosis in ascite-derived ovarian cancer cells	[70]
Bladder cancer	BCa cell organoid models	PHGDH inhibitor NCT-502 induces ferroptosis in BCa cell organoid models	[66]
Bladder cancer	Patient-derived organoids	m6A modifications affect chemoresistance by controlling SLC7A11 protein levels, influencing ferroptosis sensitivity	[71]
Cholangiocarcinoma	Patient-derived organoids	Combining SUR with PDT induces ferroptosis and inhibits tumor growth	[72]
Glioblastoma	GBM patient-derived organoid models	Combination treatment can enhance ferroptosis by regulating heme oxygenase 1 (HOXM1) and GPX4 expression	[73]
Oral squamous cell carcinoma	Patient-derived organoid models	DRP1 inhibition-mediated mitochondrial elongation drives ferroptosis and abolishes cancer stemness	[42]
Lung cancer	Lung cancer organoids	MA promotes EGFR-TKI sensitivity by the KRAS-ERK pathway and mitochondrial Ca^2+^ overload	[74]
Prostate cancer	Organoid cultures derived from prostate cancer cells	TQB3720 promotes ferroptosis through the inhibition of the AR signaling pathway	[75]
Laryngeal squamous cell carcinoma	Organoid models derived from LSCC patients	Augmented ERO1α upon mTORC1 activation induces ferroptosis resistance and tumor progression via the upregulation of SLC7A11	[76]
Head and neck cancer	HNSCC organoid models	TrxR1 induces ferroptosis and potentiates the efficacy of anti-PD-1 therapy	[77]
**Pyroptosis**			
Pancreatic and lung cancers	Patient-derived tumor organoids	Src or ceramidase inhibitor reactivates pyroptosis to reverse chemoresistance	[44]
Colorectal cancer	Colorectal cancer patient-derived organoids	BI and CPT induce GSDME-mediated pyroptosis and apoptosis, with anti-tumoural activity	[78]
Colorectal cancer	Adult stem cell-derived murine colonic epithelial organoids	EGFR-signaling-independent colonoids show chromosomal instability and altered pyroptosis-related genes	[45]
Bladder cancer	MIBC mouse model with gene-edited organoids	Cathepsin inhibition induces pyroptosis, restraining chemoresistant MIBCs	[79]
Endometrial cancer	EC-derived organoids	ERRα regulates pyroptosis through the NLRP3/caspase-1/GSDMD pathway	[80]
Esophageal adenocarcinoma	Murine BE organoids	Caspase-1 inhibition reduces IL-1β and CXCL1 secretion, linked to BE to EAC progression	[81]
Her2-positive gastric cancer	Patient-derived organoids	IBI315 induces GSDMB-mediated pyroptosis in tumor cells, enhancing T cell activation and tumor killing	[82]
Ovarian cancer	Patient-derived ovarian cancer organoids	GBP5 induces canonical pyroptosis through the JAK2/STAT1 pathway, inhibiting cancer progression	[83]
Ovarian carcinoma	Adipocyte organoid model	Adipocyte pyroptosis in an omental tumor microenvironment is associated with chemoresistance	[84]
**Necroptosis**			
Pancreatic duct adenocarcinoma	Human or mice PDAC organoids	Organoids used to study impaired cell death pathways, including necroptosis in PDAC	[85]
Colorectal cancer	Human colorectal cancer and murine organoid models	Tolinapant induces apoptosis, and its effect is augmented by FOLFOX treatment in the presence of HDAC inhibitors	[86]
Colorectal cancer	Patient-derived colon cancer organoids	FMRP regulates RIPK1 and colorectal cancer resistance to necroptosis	[49]
GVL	Mouse gastrointestinal organoids	Inhibition of RIP1 improves immune reconstitution and reduces GVHD mortality while preserving graft-versus-leukemia effects	[50]
Mutant p53-expressing cells	Intestinal organoids	Mutant p53-expressing cells undergo necroptosis via cell competition with the neighboring normal epithelial cells	[87]
Gastric cancer	Organoids derived from corpus glands	IL-17A is a cytokine that promotes parietal cell apoptosis during atrophic gastritis, a precursor lesion for gastric cancer	[88]

## Data Availability

Not applicable.

## Abbreviations

The following abbreviations are used in this manuscript:

RCD, regulated cell death; 2D, two-dimensional; 3D, three-dimensional; RIPKs, receptor-interacting protein kinases; RIPK1 and RIPK3, receptor-interacting protein kinases; ECM, extracellular matrix; System Xc-, cystine/glutamate antiporter system; GSH, glutathione; GPX4, glutathione peroxidase 4; ROS, reactive oxygen species; HIC1, hypermethylated in cancer 1; FSP1, ferroptosis suppressor protein 1; GSDMD, gasdermin D; PAMPs, pathogen-associated molecular patterns; DAMPs, damage-associated molecular patterns; PRRs, pattern recognition receptors; MLKL, pseudokinase mixed lineage kinase domain-like protein; TNFR1, tumor necrosis factor receptor-1; TNBC, triple-negative breast cancer; FAK, focal adhesion kinase; FGFR4, fibroblast growth factor receptor 4; PDAC, pancreatic ductal adenocarcinoma; MCU, mitochondrial calcium uniporter; SLC7A11, solute carrier family 7 member 11; HCC, hepatocellular carcinoma; HMOX1, heme oxygenase; PPARGC1A, peroxisome proliferator-activated receptor-gamma coactivator-1α; URI, RPB5 interactor; TKIs, tyrosine kinase inhibitors; SCD1, stearoyl-CoA desaturase 1; MDSCs, myeloid-derived suppressor cells; CRC, colorectal cancer; CAFs, cancer-associated fibroblasts; NK, natural killer; FSTL1, follistatin-like protein 1; STAT3, activator of transcription 3; MTTP, microsomal triglyceride transfer protein; LGR4, G protein-coupled receptor 4; FADS2, acyl-CoA 6-desaturase; HGSOC, high-grade serous ovarian cancer; FeNP, iron nitroprusside; PHGDH, phosphoglycerate dehydrogenase; DRP1, dynamin-related protein 1; AR, androgen receptor; ERO1α, endoplasmic reticulum oxidoreductase 1 alpha; TrxR1, thioredoxin reductase 1; ASAH2, sphingolipid metabolic enzyme ceramidase; ERRα, estrogen-related receptor alpha; GBP5, guanylate-binding protein 5; FFAs, free fatty acids; FMRP, fragile X mental retardation protein; CTSH, cathepsin H; MIBC, muscle-invasive bladder cancer; IAPs, inhibitors of apoptosis proteins; FOLFOX, fluorouracil plus leucovorin and oxaliplatin; HDAC, class I histone deacetylases; WTAP, Wilms’ tumor 1-associating protein; YTHDC2, YTH domain-containing 2.
